# Effects of dandelion root on rat heart function and oxidative status

**DOI:** 10.1186/s12906-023-03900-5

**Published:** 2023-03-10

**Authors:** Kristina Radoman, Vladimir Zivkovic, Nebojsa Zdravkovic, Natalia Vasilievna Chichkova, Sergey Bolevich, Vladimir Jakovljevic

**Affiliations:** 1 College of Health Studies, Podgorica, Montenegro; 2 Department of Physiology, Faculty of Medical Sciences, University of Kragujevac, Kragujevac, Serbia; 3 Center of Excellence for Redox Balance Research in Cardiovascular and Metabolic Disorders, Kragujevac, Serbia; 4 First Moscow State Medical University I.M. Sechenov, Moscow, Russia; 5 Department of Medical Statistics and Informatics, Faculty of Medical Sciences, University of Kragujevac, Kragujevac, Serbia; 6 Department of Faculty Therapy, First Moscow State Medical University I.M. Sechenov, Moscow, Russia; 7 Department of Human Pathology, First Moscow State Medical University I.M. Sechenov, Moscow, Russia

**Keywords:** Dandelion root, Cardiodynamics, Oxidative stress, Rat

## Abstract

This study aimed to examine the effects of dandelion root on rat heart function and oxidative status. At the beginning of the experimental protocol, *Wistar albino* rats were randomly classified into two groups (10 rats per group): 1. control group – animals that drank tap water; 2. experimental group – animals that drank dandelion root for four weeks. Every morning for four weeks, the animals received freshly boiled dandelion root in a volume of 250 ml. At the end of the dandelion administration, animals were sacrificed, and their hearts were isolated and retrogradely perfused according to the Langendorff technique at a gradually increasing perfusion pressure between 40 – 120 cm H_2_O. The following myocardial function parameters were measured: maximum rate of left ventricular pressure development (dp/dt max), minimum rate of left ventricular pressure development (dp/dt min), systolic left ventricular pressure (SLVP), diastolic left ventricular pressure (DLVP), heart rate (HR). In addition, the coronary flow (CF) was measured flowmetrically. Finally, blood samples were collected after sacrificing to determine oxidative stress biomarkers: nitrite (NO_2_^−^), superoxide anion radical (O_2_^−^), hydrogen peroxide (H_2_O_2_), the index of lipid peroxidation (TBARS), reduced glutathione (GSH), catalase (CAT) and superoxide dismutase (SOD). The present pioneer results indicated that dandelion root did not manifest a negative impact on functional aspects of isolated rat heart. In addition, dandelion consumption was not associated with promising results in terms of maintaining systemic redox balance.

## Introduction

Dandelion *(Taraxacum officinale Weber)* belongs to the *Taraxacum* genus, a member of the *Asteraceae* family and *Cichoriodeae* subfamily. It is widespread in the warm and humid zones characteristic of the northern hemisphere and has long been used in traditional medicine in the form of infusions and decoctions [1].

The medicinal raw materials of this plant consist of roots, leaves, and flowers. Environmental conditions, periods of plucking, different plucking methods, and drying methods significantly influence the chemical composition of the materials themselves. Dandelions are a rich source of various phyto compounds such as flavonoids, phenolic acids, terpenes, and polyphenolic compounds [2, 3]. The positive health effects of dandelions are a consequence of their phytochemical properties.

Due to such a composition, they exhibit potent antioxidant and anti-inflammatory effects. Thus, it has been observed that treating mice with a herbal mixture containing dandelion can cause a decrease in lipid peroxidation in serum and tissues and increase the activity of antioxidant protection enzymes (superoxide dismutase peroxidase and reduced glutathione) [4]. In addition, it has been observed in vitro and in vivo that dandelion can suppress the production of tumor necrosis factor (TNF-α) and interleukin-6 (IL-6). The animal model of diabetes mellitus–induced renal injury can be successfully treated with dandelion by reducing the production of interleukin-6 and TNF-α [5].

Although this plant is well known in traditional herbal medicine, there is only limited relevant scientific information on its pharmacological effects, with often contradictory results [6]. Recent research has presented dandelion as a new candidate for the fight against cancer since the aqueous extract from dandelion root has shown antineoplastic effects on aggressive and resistant cells of chronic myelomonocytic leukemia (CML) [7].

On the other hand, the cardiovascular effects of dandelion have been increasingly studied in recent years. They are based on its antiatherosclerotic potential resulting from antioxidant and anti-inflammatory properties. Recent experimental studies have shown that treatments with various dandelion extracts reduce adipogenesis and lipid accumulation, severity of atherosclerosis, serum concentrations of total cholesterol, triglycerides, and LDL cholesterol with an increase in HDL cholesterol [8–10].

However, despite the promising effects of dandelion in treating and preventing cardiovascular diseases, there is almost no data on the impact of this plant’s extracts on the heart. In that sense, this study aimed to examine the effects of dandelion root on rat heart function and oxidative status.

## Materials and methods

### Ethical aspects

This investigation was conducted in the Laboratory for cardiovascular physiology of the Faculty of Medical Sciences, University of Kragujevac, Serbia. The study protocol was approved by the Ethical Committee for the welfare of experimental animals of the Faculty of Medical Sciences, University of Kragujevac, Serbia. All experiments were performed following ARRIVE guidelines 2.0 for reporting animal research.

### Reagents

All reagents used in this study were of high purity and manufactured by Sigma-Aldrich Chemie GmbH, Germany.

### Experimental animals and groups

The study was carried out on 20 male *Wistar albino* rats (8 weeks old, body weight 250 ± 20 g). The animals consumed commercial rat food (20% protein rat food, Veterinary Institute Subotica, Serbia) and were housed under controlled environmental conditions at room temperature (22 ± 1 °C) with a 12-h light/day photoperiod. The rats had free access to food and tap water ad libitum. At the beginning of the experimental protocol, rats were randomly classified into two groups (10 rats per group):

1. Control group – animals that drank tap water.
2. Experimental group – animals that drank dandelion root for four weeks [6].

### Preparation of dandelion root

A total of 3 g chopped dandelion root is added to 300 ml of cold water, then heated and boiled for 5 min. After boiling, the root is left for ten minutes to cool and then filtered.

### Experimental protocol

Every morning for four weeks, the animals received fresh dandelion root in a 250 ml volume bottle [6]. In order to accurately record the intake of dandelion root, each animal was placed in a separate cage, while the volume of tea was recorded daily. The average daily dandelion root intake was 39.44 ± 2.67 ml in the experimental group, while the control group took tap water in an average amount of 42.85 ± 3.16 ml.

The animals were subjected to anesthesia at the end of the experimental protocol prior to sacrifice. A mixture of ketamine (Vet-Agro, Lublin, Poland) and xylazine (De Adelaar B.V, Venray, Holland) was prepared in a syringe. Administration of 25 µl/kg ketamine and 62.5 µl/kg xylazine was equivalent to the recommended dosage of 10 mg ketamine/kg and 5 mg xylazine/kg for rats [11]. The ketamine/xylazine mixture was administered i.p., and after 2 min, animals were sacrificed by decapitation.

### Evaluation ofex vivocardiac function

After decapitation, an emergency thoracotomy was performed, and rat hearts were isolated, attached via an aortic cannula, and retrogradely perfused using the Langendorff technique at a gradually increasing perfusion pressure between 40 – 120 cm H_2_O [12]. The hearts were perfused with Krebs–Henseleit solution (118 mM NaCl, 4.7 mM KCl, 2.5 mM CaCl_2_ 2H_2_O, 1.7 mM MgSO_4_ H_2_O, 25 mM NaHCO_3_, 1.2 mM KH_2_PO_4_, 5.5 mM glucose, equilibrated with 95% O_2_/5% CO_2_) and warmed to 37 °C (pH = 7.4). After heart perfusion commenced, a 30-min period was allowed for the hearts to stabilize. A transducer (BS473-0184, Experimetria Ltd., Budapest, Hungary) was used to monitor the following parameters of myocardial function: maximum rate of left ventricular pressure development (dp/dt max), minimum rate of left ventricular pressure development (dp/dt min), systolic left ventricular pressure (SLVP), diastolic left ventricular pressure (DLVP), heart rate (HR). The coronary flow (CF) was measured flowmetrically.

### Biochemical assay in blood

Blood samples were collected after decapitation in a vacutainer tube containing EDTA as an anticoagulant for the assay of pro-oxidative markers in the plasma and antioxidant markers in the lysate. The samples were centrifuged at 3000 rpm for 10 min at 4 °C using a Centurion centrifuge (K280R, UK). The plasma and erythrocyte lysate were then stored at -20 °C until analysis. All measurements were performed spectrophotometrically (Shimadzu UV-1800, Japan).

### Determination of oxidative status markers in blood

In plasma samples, the following oxidative stress markers were measured: nitrite (NO_2_), superoxide anion radical (O_2_^−^), hydrogen peroxide (H_2_O_2_), and the index of lipid peroxidation (measured as TBARS – thiobarbituric acid reactive substances).

Nitric oxide decomposes rapidly to form stable metabolite nitrite/nitrate products. The nitrite level was measured and used as an index of nitric oxide (NO) production using the Griess reagent. A total of 0.5 ml of plasma was precipitated with 200 μl of 30% sulphosalicylic acid, vortexed for 30 min, and centrifuged at 3000 × g. Equal volumes of supernatant and Griess reagent containing 1% sulphanilamide in 5% phosphoric acid/0.1% naphthalene ethylenediamine dihydrochloride were added and incubated for 10 min in the dark, and the sample was measured at 543 nm. The nitrite levels were calculated using sodium nitrite as the standard [13].

The O_2_^−^ concentration was measured after the reaction of nitro blue tetrazolium in Tris buffer with the plasma at 530 nm. Distilled water served as the blank [14].

The measurement of H_2_O_2_ is based on the oxidation of phenol red by H_2_O_2_ in a reaction catalysed by horseradish peroxidase (HRPO). Two hundred μl of plasma was precipitated with 800 ml of freshly prepared phenol red solution, followed by the addition of 10 μl of (1:20) HRPO (made ex tempore). Distilled water was used as the blank instead of the plasma sample. H_2_O_2_ was measured at 610 nm [15].

The degree of lipid peroxidation in the plasma samples was estimated by measuring TBARS using 1% thiobarbituric acid in 0.05 NaOH, incubated with the plasma at 100 °C for 15 min, and measured at 530 nm. Distilled water served as the blank [16].

The activity of the following antioxidants in the lysate was determined: reduced glutathione (GSH), catalase (CAT), and superoxide dismutase (SOD). The level of reduced glutathione was determined based on GSH oxidation with 5,5-dithiobis-6,2-nitrobenzoic acid using a method by Beutler [17]. The CAT activity was determined according to Aebi [18]. The lysates were diluted with distilled water (1:7 v/v) and treated with chloroform-ethanol (0.6:1 v/v) to remove haemoglobin, and then 50 μl of CAT buffer, 100 μl of sample and 1 ml of 10 mM H_2_O_2_ were added to the samples. The detection was performed at 360 nm. SOD activity was determined by the epinephrine method of Beutler [19]. Lysate (100 μl) and 1 ml carbonate buffer were mixed, and then 100 μl of epinephrine was added. The detection was performed at 470 nm.

### Statistical analysis

The collected data were processed using the SPSS 21.0 software (SPSS Inc., Chicago, IL, USA). The Shapiro–Wilk test was used to examine data distribution normality. Depending on the data distribution, Student’s t-test and Kruskal–Wallis tests were applied to analyse parametric and nonparametric data, respectively. The confidence interval in all statistical analyzes is 95%, with a statistical significance *p* < 0.05 and a high statistical significance *p* < 0.01. Data are described as mean ± standard deviation (SD).

## Results

### Ex vivoparameters of cardiac function

**Fig. 1:**
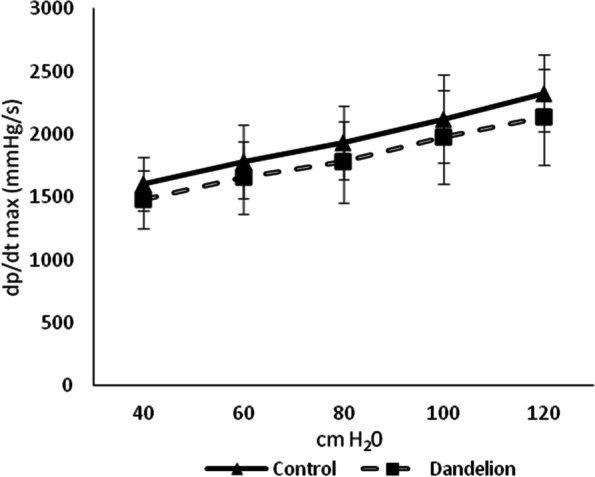
Average values ​​of the maximum rate of pressure change in the left ventricle (dp/dt max (mmHg/s)) in the control and dandelion infusion-treated groups. Data is presented in the form of mean value ± SD

**Fig. 2:**
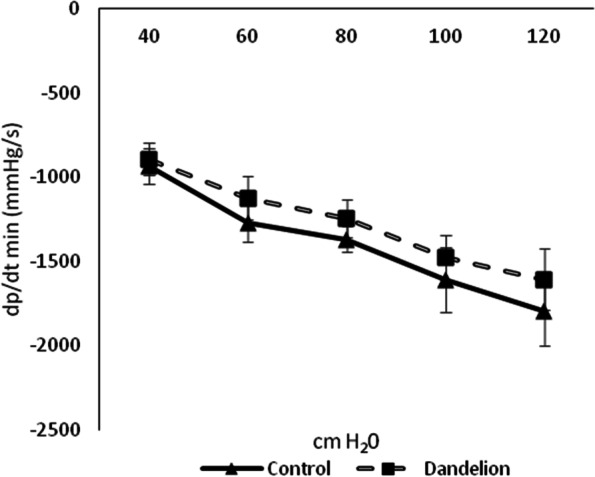
Average values ​​of the minimum rate of pressure change in the left ventricle (dp/dt min (mmHg/s)) in the control and dandelion infusion-treated groups. Data is presented in the form of mean value ± SD

The mean values of the maximum rate of change in left ventricular pressure (dp / dt max) did not differ statistically significantly (*p* > 0.05) between the control and experimental group at all values of coronary perfusion pressure (Fig. 1). The mean values of the minimum rate of change in left ventricular pressure (dp / dt min) did not differ significantly (*p* > 0.05) between the control and experimental group at all values of coronary perfusion pressure (Fig. 2).

**Fig. 3:**
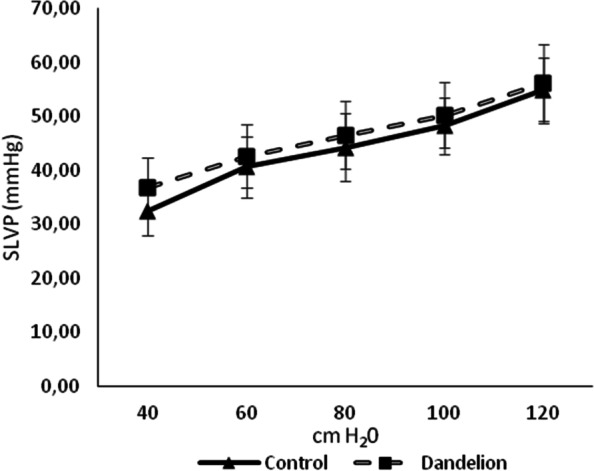
Average values ​​of systolic left ventricular pressure (SLVP (mmHg)) in the control and dandelion infusion-treated groups. Data is presented in the form of mean value ± SD

**Fig. 4:**
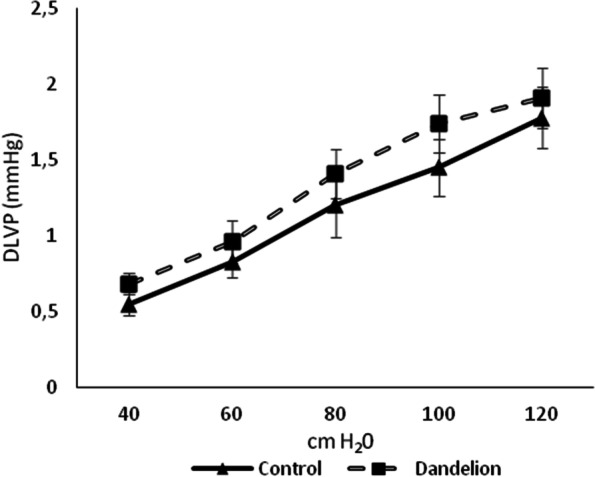
Average values ​​of diastolic left ventricular pressure (DLVP (mmHg)) in the control and dandelion infusion-treated groups. Data is presented in the form of mean value ± SD

**Fig. 5:**
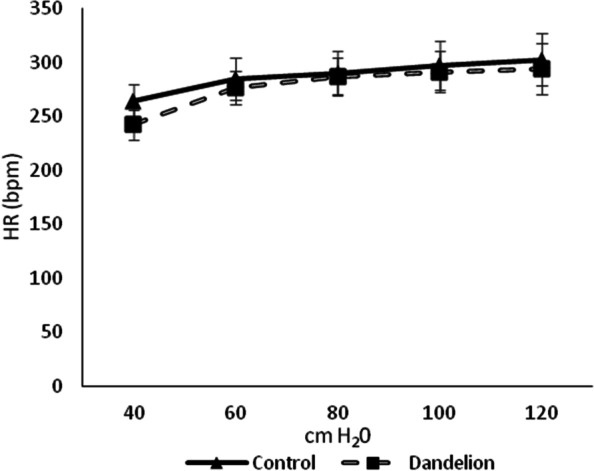
Average values ​​of heart rate (HR (bpm)) in the control and dandelion infusion-treated groups. Data is presented in the form of mean value ± SD

The average values of systolic and diastolic pressure in the left ventricle (SLVP and DLVP) did not differ statistically significantly (*p* > 0.05) between the dandelion-treated and control group (Figs. 3 and 4). The mean values of the heart rate (HR) were very close between the groups, thus there was no statistical difference in this case either (*p* > 0.05) (Fig. 5).

**Fig. 6:**
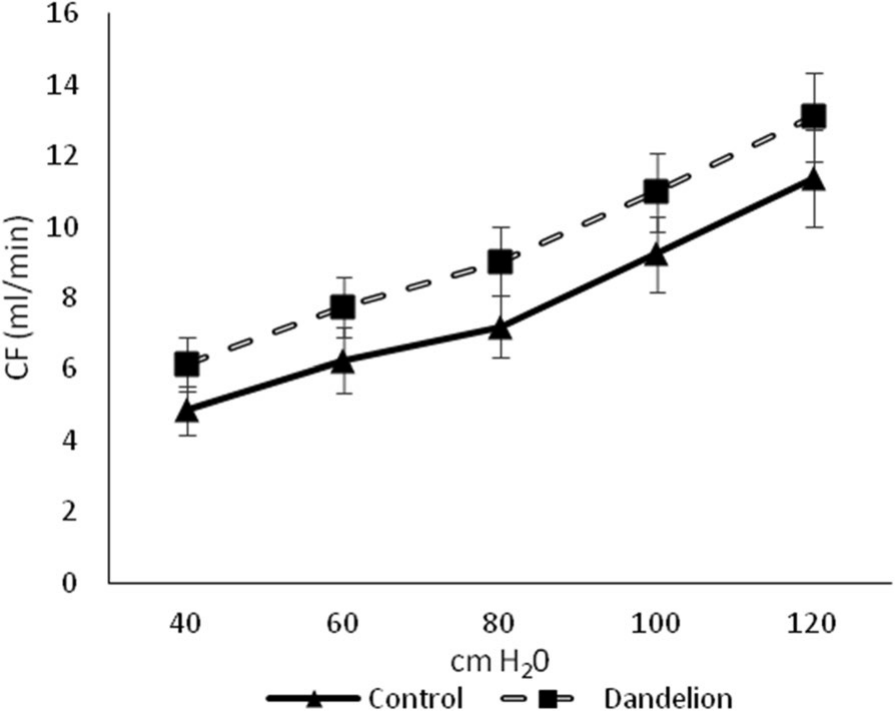
Average values ​​of coronary flow (CF (ml/min)) in the control and dandelion infusion-treated groups. Data is presented in the form of mean value ± SD

The mean values of coronary flow during all values of coronary perfusion pressure were higher in the group that was administered dandelion root but without statistical confirmation (*p* > 0.05) (Fig. 6).

### Oxidative status markers in blood

**Fig. 7:**
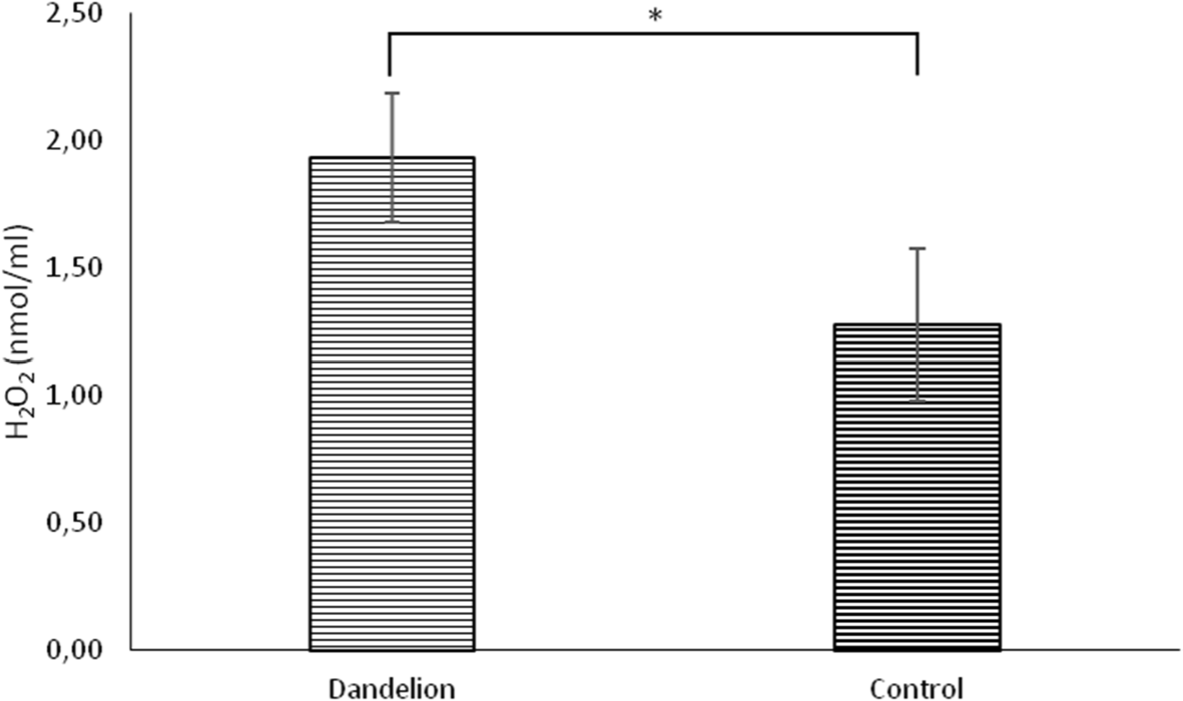
Average values ​​of the concentration of hydrogen peroxide (H_2_O_2_ (nmol/ml)) in the control and dandelion infusion-treated groups. Data is presented in the form of mean value ± SD. *—statistically significant, *p* < 0.05

**Fig. 8:**
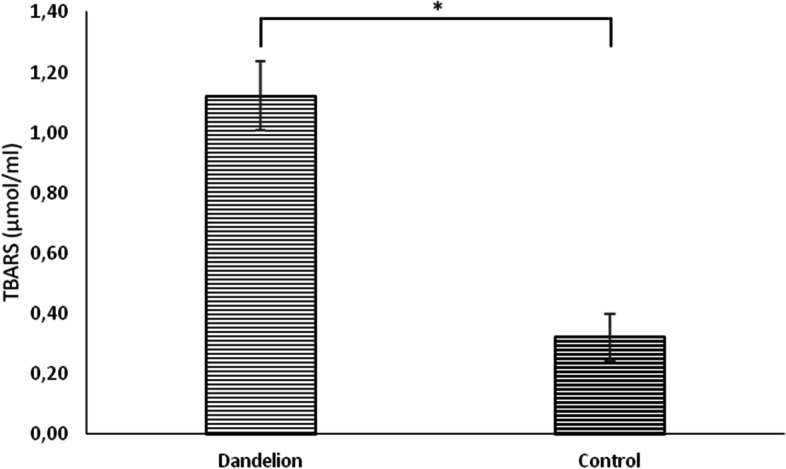
Average values ​​of the concentration of thiobarbituric acid reactive substances (TBARS (µmol/ml)) in the control and dandelion infusion-treated groups. Data is presented in the form of mean value ± SD. *—statistically significant, *p* < 0.05

**Fig. 9:**
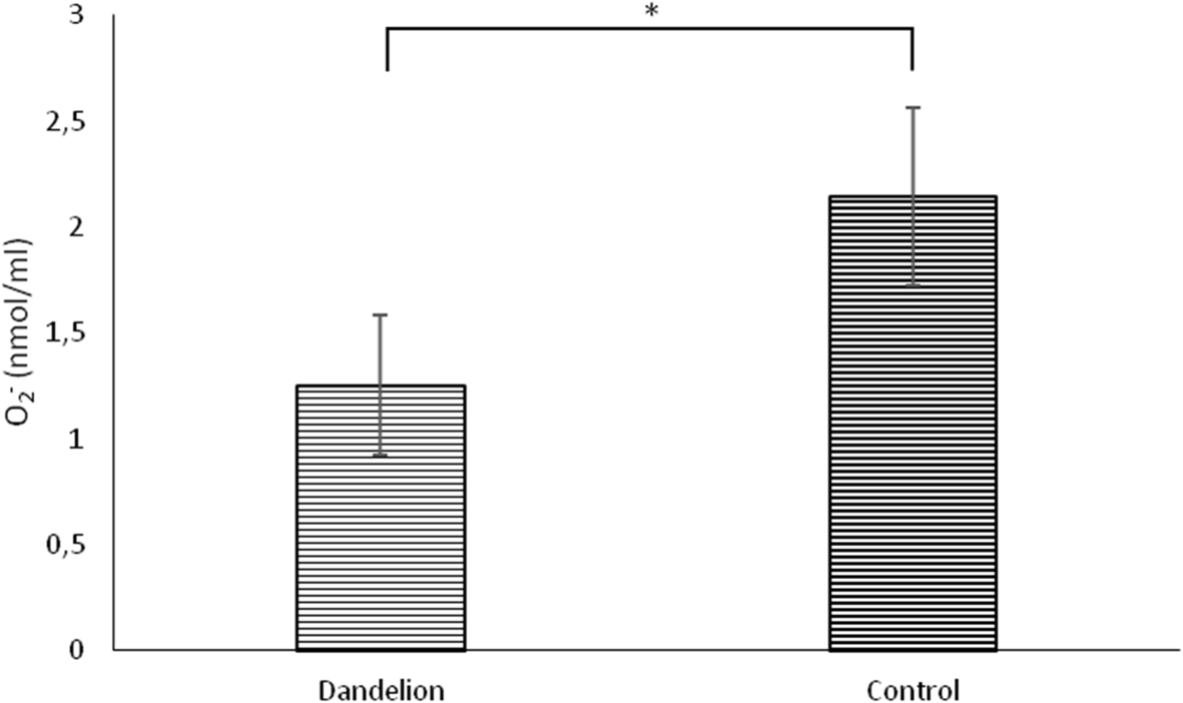
Average values ​​of the concentration of superoxide ion radical (O_2_.^−^ (nmol/ml)) in the control and dandelion infusion-treated groups. Data is presented in the form of mean value ± SD. *—statistically significant, *p* < 0.05

In rats treated with dandelion root, there was a statistically significant increase (*p* < 0.05) in hydrogen peroxide (H_2_O_2_) values compared to the control group (Fig. 7). Lipid peroxidation index (TBARS) showed similar dynamics, whose values ​​were significantly higher (*p* < 0.05) in the experimental group compared to the control group (Fig. 8). The superoxide anion radical (O_2_^−^) values ​​were statistically significantly lower (*p* < 0.05) in the rats on dandelion consumption than in the control group (Fig. 9).

**Fig. 10:**
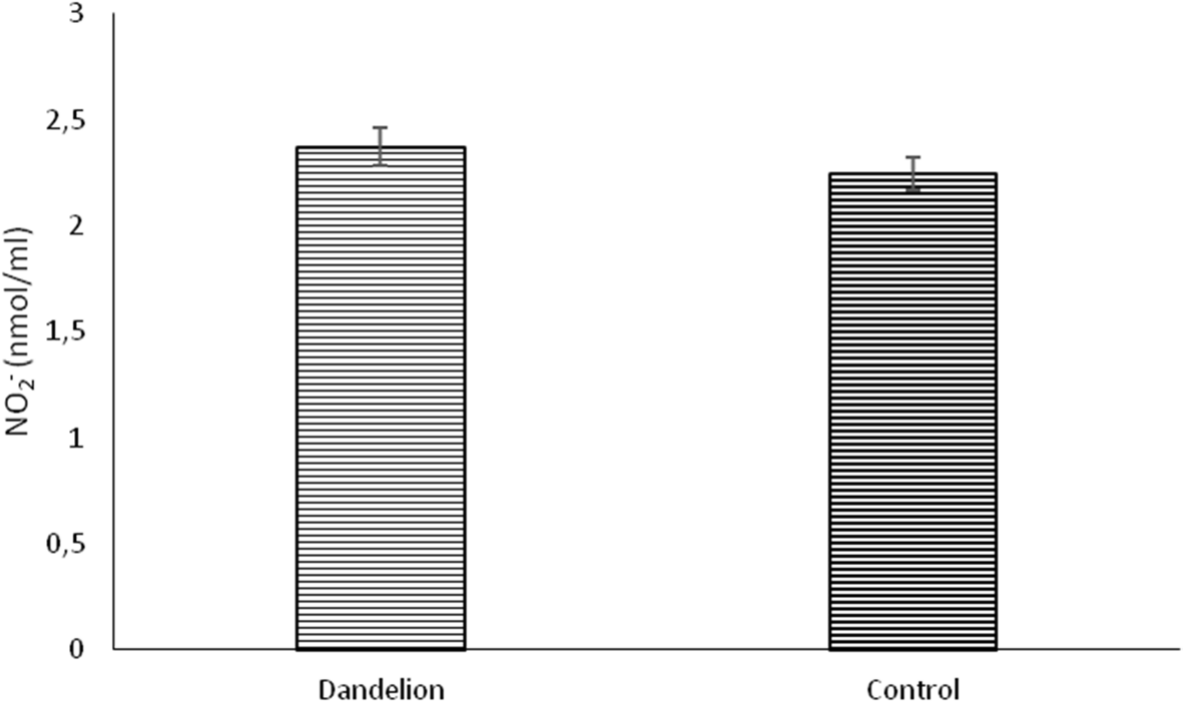
Average values ​​of the concentration of nitrite ions (NO_2_^−^ (nmol/ml)) in the control and dandelion infusion-treated groups. Data is presented in the form of mean value ± SD

**Fig. 11:**
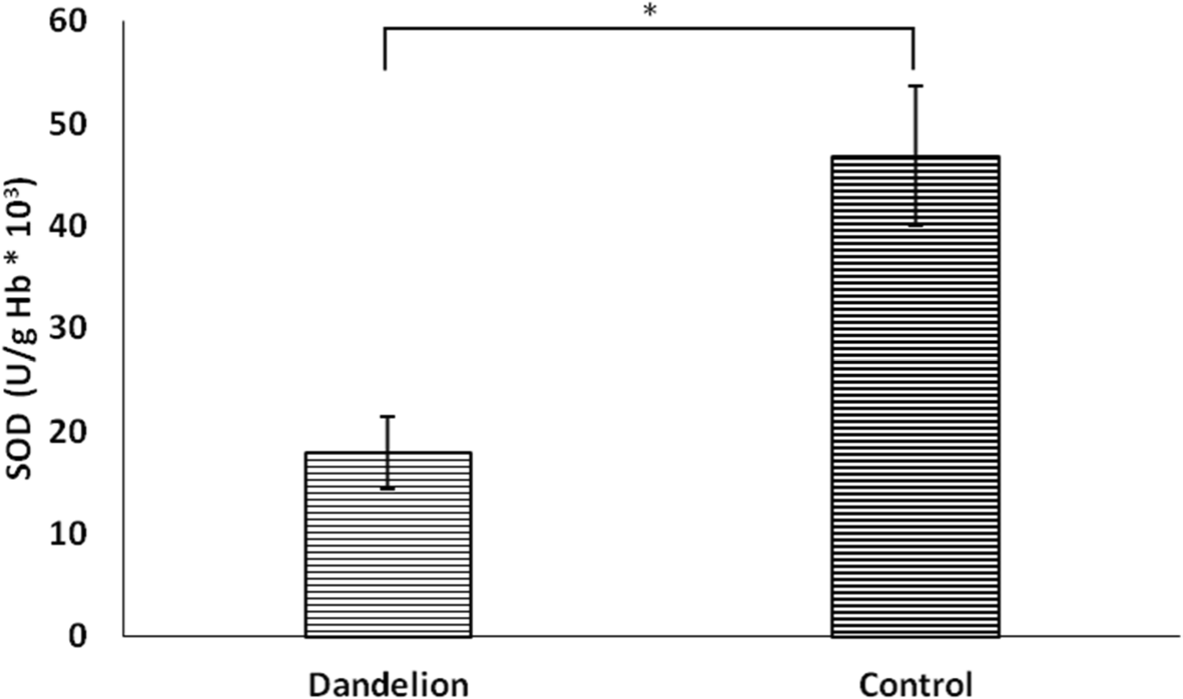
Average values ​​of the concentration of superoxide dismutase (SOD (U/g Hb*10.^3^)) in the control and dandelion infusion-treated groups. Data is presented in the form of mean value ± SD. *—statistically significant, *p* < 0.05

**Fig. 12:**
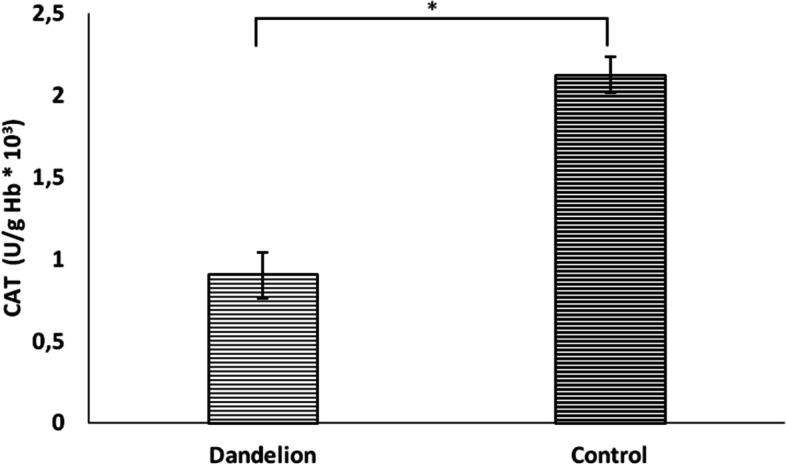
Average values ​​of the concentration of the catalase enzyme (CAT (U/g Hb*10.^3^)) in the control and dandelion infusion-treated groups. Data is presented in the form of mean value ± SD. *—statistically significant, *p* < 0.05

**Fig. 13:**
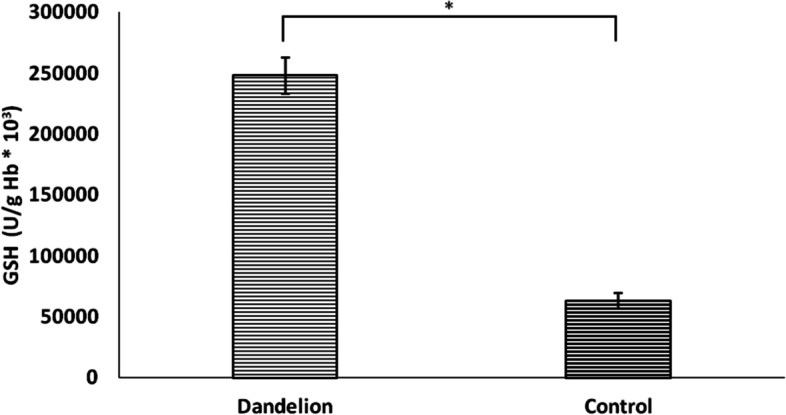
Average values ​​of the concentration of glutathione (GSH (U/g Hb*10.^3^)) in the control and dandelion infusion-treated groups. Data is presented in the form of mean value ± SD. *—statistically significant, *p* < 0.05

Unlike previous biomarkers, nitrite (NO_2_^−^) values ​​did not differ statistically significantly between groups (*p* > 0.05) (Fig. 10). On the other hand, catalase and superoxide dismutase activity was significantly lower (*p* < 0.05) in the dandelion-treated group (Figs. 11 and 12). In comparison, reduced glutathione activity was statistically higher (Fig. 13) than in the control group (*p* < 0.05).

## Discussion

The main goal of the present study was to examine the effect of dandelion root on rat heart function and oxidative status.

In the first part of the study, the effect of dandelion root on the cardiac muscle was studied through an assessment of the cardiodynamic parameters of an isolated rat heart. As previously mentioned, to our best knowledge, this is one of the rare studies covering this topic in the available literature. Therefore, we can consider this a "pioneering" investigation, i.e., a topic that requires further research.

The beneficial effects of dandelion in maintaining cardiovascular homeostasis have been known for a long time in traditional and folk medicine [9, 10]. However, in recent years investigations have become more complex and focused on different aspects of this field. The study by Majewski et al. was primarily concerned with examining the effect of plant extract on rat antioxidant status and lipid profile [20]. The single cardiodynamic parameter analyzed in this study was heart rate (HR). As in the present research, authors also used 8-week-old *Wistar albino* rats. The differences between the protocols are reflected in the fact that Majewski et al. used an ethanolic extract of dandelion leaves and flowers [20].

In contrast, we used an aqueous extract of dandelion root. The reason for using a root is our literature-based hypothesis that its phytochemical properties remain more stable when tea is made from this plant part [2, 3].

The results of this study, in terms of heart rate (HR), are in agreement with those of the above study. In both cases, there was no evident change in heart frequency after applying the dandelion extract, suggesting that dandelion root does not modify the function of the heart conduction system.

Langendorff preparation optimizes the detection of ventricular function and permits exceptionally accurate measurement intervals [21]. Indicators of contractile (dp / dt max) and relaxant (dp / dt min) force of the heart were also not affected by dandelion root. Similarly, dandelion did not impair systolic (SLVP) and diastolic (DLVP) function of the cardiac muscle as well as reactivity of coronary circulation (CF). Taken together, these results indicated that applied dandelion root did not negatively impact the function and perfusion of isolated rat heart. However, the exact mechanism of these effects requires a more complex experimental approach. In addition, considering the steady trends of non-statistical cardiodynamic changes, it should be pointed out that higher doses or extended time of exposure seem to be associated with different findings, which imposes the need for further research.

One of the rare studies of the influence of dandelion on any muscle was recently published [22]. Namely, ethyl acetate dandelion extract’s effects were evaluated on mouse airway smooth muscle. This extract was found to relax mouse smooth muscle via inhibition of L-type voltage-dependent calcium channel and non-selective cationic channel, which, at least theoretically, could be the site of action in rat cardiomyocytes [22].

On the other hand, in the second part of the research, we seek to examine whether, taking into account the antioxidant properties of dandelion, it can disrupt the redox homeostasis of rats, which can also be responsible for the obtained effects within the heart. It is well known that the accumulation of reactive oxygen species (ROS) leads to biochemical, structural, and functional disorders in cells [23]. It has been proven that dandelion successfully prevents synthesis and increases the removal of different ROS, especially H_2_O_2_ and O_2_^−^. The capacity to remove free radicals has been attributed to phenolic compounds in dandelion flowers [24].

Dandelion is widely used as a folk remedy against various disorders such as liver disease, bile, indigestion, and rheumatic diseases. In one research, dandelion leaf has been shown to possess a protective effect against acute pancreatitis caused by cholecystokinin octapeptide and acute lung and liver damage. This protective effect is due to components from dandelion leaves with flavonoids and polyphenols [25]. Plant flavonoids act as scavengers of free radicals and turn them into less reactive or bind metal ions preventing their production [26].

In our study, dandelion root affected the production of measured pro-oxidants oppositely. Namely, while the release of O_2_^−^ was decreased, the concentration of H_2_O_2_ and TBARS were higher than in the control group. The explanation for this different impact on ROS generation from the point of this study is difficult to find. Literature data show that dandelion lowers the concentration of TBARS and H_2_O_2_, but only in in vitro conditions [27], while there are no data related to in vivo biological systems. It seems that the well-known antioxidant effects of dandelion directed towards individual ROS can only be achieved in cell lines, while in vivo systems require a higher dose or length of exposure to achieve them. In addition, the worrying increase of both investigated biomarkers after the use of dandelion (TBARS and H_2_O_2_) may be a consequence of the compensatory overproduction of other ROS in a situation where O_2_^−^ generation is suppressed [23]. Nevertheless, the reduction of O_2_^−^ we noted is a promising finding since it is one of the most toxic known ROS and completely correlates with literature findings [24, 25].

In addition, the concentrations of free radicals depend not only on their production but also on the expression and activity of antioxidant enzymes [28]. Flavonoids found in the dandelion extract have a beneficial effect on cardiovascular function based on their antioxidant features and the ability to increase the expression and activity of antioxidant enzymes [29, 30].

In the present investigation, the four-week dandelion consumption also impacted estimated antioxidant enzymes differently. While CAT and SOD activity was lower, reduced glutathione activity was improved compared to control. The drop in CAT values may be a reflection of the exhaustion of its activity as a consequence of the increased release of H_2_O_2_ we found. Furthermore, previous research examined the hypolipidemic and antioxidant potential of animals treated with dandelion leaf and root extract [31]. The activity of reduced glutathione was strongly improved in the group treated with dandelion root and leaf compared to the control group, while catalase activity was lower, which is in complete correlation with the results of our study [31].

A study by Park and associates compared the antioxidant and anti-inflammatory activity of methanolic and aqueous extracts of *Taraxacum officinale.* The activity of reduced glutathione and other antioxidant enzymes such as superoxide dismutase, catalase, glutathione peroxidase, and glutathione reductase were restored after using the extract. Methanol extract showed more potent antioxidant and anti-inflammatory abilities than aqueous extract, which can be attributed to the higher total content of phenol, luteolin, and cichoric acid [32].

Finally, the present research has some limitations. First, the longer duration of dandelion consumption and assessment of different doses could have a stronger impact on both heart and oxidative status of rats. Second, due to technical limitations, the study was limited in mechanistic approach, i.e., patch clamp assessment of ionic currents within the cardiomyocytes would provide potential mechanisms of dandelion effects. Pathohistological examination of heart tissue can also serve this purpose.

## Conclusion

To the best of our knowledge, this is the only experimental study investigating the effects of dandelion root on the mammalian heart and oxidative status in the available literature. The present pioneer results indicated that dandelion root did not manifest a negative impact on functional aspects of isolated rat heart. In addition, the impact of dandelion root on systemic oxidative status was variable and individually directed toward measured biomarkers. Therefore the global systemic antioxidant effect was not achieved. From a clinical perspective, these findings may be an excellent basis as the first step in developing an animal model of heart failure or other cardiovascular disease, where dandelion usage may have a practical benefit.

## Data Availability

The datasets generated and analysed during the current study are not publicly available due to confidentiality and safety reasons but are available from the corresponding author upon reasonable request.
